# Fifth‐Time Recurrence of Dermatofibrosarcoma Protuberans at Distinct Sites: A Rare Case Report

**DOI:** 10.1002/ccr3.71636

**Published:** 2025-12-21

**Authors:** Hamna Tariq, Hadiya Javed, Mohammed Hammad Jaber Amin

**Affiliations:** ^1^ Department of Surgery Dow Medical College Karachi Pakistan; ^2^ Department of Medicine Alzaiem Alazhari University Khartoum Sudan

**Keywords:** dermatofibrosarcoma protuberans, fibrosarcoma, neoplasm recurrence, sarcoma, skin neoplasms

## Abstract

Dermatofibrosarcoma protuberans is a rare dermal sarcoma with high local recurrence and rare metastasis. Our case highlights unusually frequent recurrences at different trunk sites over 8 years, including fibrosarcomatous transformation and nodal metastasis, underscoring the need for vigilant long‐term surveillance and tailored management.

## 1 Introduction

Dermatofibrosarcoma protuberans (DFSP) is a rare dermal‐based sarcoma that originates in the dermis and extends into the underlying tissues [1]. DFSP accounts for approximately 1.0% of all soft tissue sarcomas, with an annual incidence estimated at 0.8–5 cases per million population. It is most commonly diagnosed in patients aged 20–59 years [2]. The incidence varies by sex and race, occurring twice as frequently in women as in men and twice as commonly in Black individuals compared to White individuals [3].

The etiology of DFSP remains incompletely understood; however, approximately 10% of cases have a history of prior trauma, including surgical scars, burns, or immunization at the affected site [3]. The pathophysiology of DFSP is driven by a characteristic chromosomal translocation, *t*(17;22), resulting in the fusion of the collagen type I alpha 1 (COL1A1) and platelet‐derived growth factor subunit B (PDGFB) genes. This fusion protein leads to overactivation of the PDGF receptor beta (PDGFRB) tyrosine kinase, promoting tumorigenesis through autocrine and paracrine stimulation of PDGF signaling [4]. Clinically, 50%–60% of DFSP lesions occur on the trunk, 25% on the upper limbs, and 10%–15% on the head and neck [4].

DFSP is characterized by a high rate of local recurrence but a low metastatic potential [5].

Although it is generally classified as a low‐grade malignancy, 10%–20% of tumors may undergo high‐grade histologic transformation, increasing the risk of local recurrence and distant metastasis [6, 7]. Complete surgical excision with microscopically negative margins remains the primary treatment for localized DFSP. Adjuvant radiotherapy is indicated in cases with positive margins or unresectable tumors. Recurrence is often attributable to residual microscopic tumor projections at the surgical margins [3].

Here, we present a case of fifth‐time recurrent dermatofibrosarcoma protuberans in a 45‐year‐old male patient. This case report has been prepared in accordance with the SCARE 2023 Criteria [8].

## 2 Case History and Examination

A 45‐year‐old male with no known co‐morbidities and a proven case of fifth‐time recurring dermatofibrosarcoma of the abdominal wall presented with the complaint of right‐sided abdominal swelling. The patient was in a normal state of health until 2016, when he first developed a pea‐sized swelling in his right lower quadrant, which upon histology, proved to be dermatofibrosarcoma protuberans. The patient was asked to follow up. In 2018, the patient developed another lesion medially on the right side of the abdomen, which was diagnosed as dermatofibrosarcoma and was surgically excised.

In 2021, the patient developed another 90 mm swelling on the right side of the abdomen. An MRI was done which showed muscle involvement. A biopsy of a 12 × 12 × 6 cm specimen partly covered by an ellipse of skin (12 × 5.5 cm) showed a gray brown nodulated mass 10 cm in diameter, reaching close to the deeper surfaces of the specimen. Upon microscopy, the skin underneath fibroadipose tissue showed infiltrating spindle cells arranged in fascicles and a herringbone pattern; the specimen showed 6–8 mitoses/10 HPF along with areas of necrosis. Histopathology led to the diagnosis of dermatofibrosarcoma protuberans, reaching close to the deeper surface. The lesion was surgically removed. A CT scan done post‐surgery did not show any residual or recurrent mass or any metastasis.

In 2023, the disease occurred for the 4th time with the lesion measuring 6.5 × 5.5 × 2 cm. A biopsy was performed which showed infiltrating spindle cells arranged in fascicles in a storiform pattern. The cells were hyperchromatic with pleomorphic nuclei and frequent mitosis (> 20/10 HPF). No necrosis was seen; however, there were intermixed ecstatic vessels. A diagnosis of fibrosarcoma GII was made based upon the histology and the tumor was surgically excised.

In 2024, the patient developed two new large lesions on the right abdominal wall. On examination, both lesions were palpable in the epigastric region and the right lower quadrant, respectively. Gut sounds were also audible. The description of the lesions is as follows: a 9.7 × 12 × 13.2 cm cystic lesion encasing the 10th and 11th ribs and posteriorly protruding into the abdomen, displacing the underlying bowel loop, and an 8.0 × 6.8 × 5.0 mm inferolaterally associated with diffuse surrounding soft tissue thickening and subcutaneous fat standing on the right‐sided abdominal wall (Table 1).

**TABLE 1:** Summary of DFSP recurrences.

	Year	Location	Size	Histology	Margins	Treatment
First occurrence	2016	Right lower quadrant, abdominal wall	Pea‐sized (few mm)	DFSP	Not specified	WLE
Second occurrence	2018	Medial right abdominal wall	Tennis‐ball sized (6–7 cm)	DFSP	Not specified	WLE
Third occurrence	2021	Right abdominal wall (muscle involvement)	12 × 12 × 6 cm	DFSP, spindle cells in fascicles & herringbone pattern, mitoses 6–8/10 HPF, necrosis present	Tumor reaching close to deeper surface	WLE
Fourth occurrence	2023	Right abdominal wall	6.5 × 5.5 × 2 cm	Fibrosarcoma Grade II, spindle cells in fascicles, storiform pattern, pleomorphic nuclei, > 20 mitoses/10 HPF, no necrosis	Not specified	WLE
Fifth occurrence	2024	Right abdominal wall—epigastric region (lesion 1), right lower quadrant (lesion 2)	Lesion 1: 9.7 × 12 × 13.2 cm Lesion 2: 8.0 × 6.8 × 5.0 cm	Low‐grade fibrosarcoma recurrent, spindle/plump cells, prominent nucleoli, ~15 mitoses/10 HPF, thin‐walled vessels	Not specified	Planned WLE, no systemic therapy given

## 3 Methods

The CT scan of the abdomen was performed which showed enlarged lymph nodes measuring 2.5 × 0.8 cm along the right distal external iliac artery and a few enhancing lymph nodes in the inguinal and axillary regions. A calcified hemangioma was also observed in the L4 vertebral body. No pulmonary, hepatic, or adrenal metastasis was seen. Upon biopsy, sections showed an ill‐defined cell lesion made of spindle to plump cells in fascicles and sheets with prominent nucleoli and up to 15 mitoses/10 HPF. Thin‐walled vessels were also present. Skeletal scintigraphy was performed to see the extent of bone metastasis with Tc‐99m MDP. 18mCi of the radiotracer was injected intravenously and whole body static images were obtained 3 h p.i. in anterior and posterior positions. The scintigraphy scans showed non‐homogeneously increased radiotracer uptake over bilateral shoulder and knee joints, indicating arthritis. The rest of the scan showed no evidence of skeletal metastasis. A low‐grade fibrosarcoma recurrent was diagnosed and the patient was advised surgical referral.

## 4 Conclusions and Results

The patient has a history of 4 surgical procedures, all proven cases of dermatofibrosarcoma. The lesions occurred at different sites each time. The treatment approach has been a wide local excision every time; no radiation or chemotherapy has been given. This clinical case emphasizes the importance of early diagnosis, as DFSP is a slow‐growing and clinically asymptomatic tumor. These characteristics delay diagnosis, allowing the tumor to penetrate deep into the tissues which ultimately require broad and deep excision that can affect cosmetic appearance. The five‐time recurrence in different sites, each time with no history of local recurrences after undergoing wide lesion excision (WLE), deviates from the usual pattern. Additionally, the frequent recurrences within a short span, the absence of metastasis when the lesion was confirmed FS and subsequent rare nodal metastasis are all highly unusual presentations.

## 5 Discussion

**FIGURE 1:**
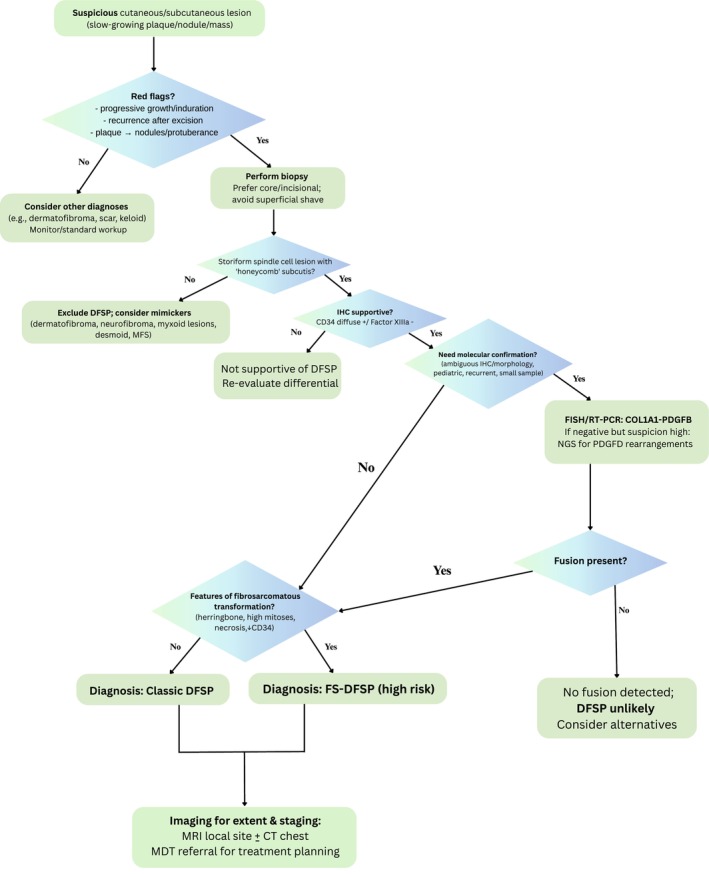
Diagnostic algorithm for DFSP and related skin conditions. DFSP, Dermatofibrosarcoma protuberans; FISH, Fluorescence In Situ Hybridization; FS‐DFSP, Fibrosarcomatous Dermatofibrosarcoma Protuberans; IHC, Immunohistochemistry; MDT, Multidisciplinary Team; MFS, Myxofibrosarcoma; NGS, Next‐Generation Sequencing; RT‐PCR, Reverse Transciption Polymerase Chain Reaction.

Dermatofibrosarcoma protuberans (DFSP) is the most common form of dermal sarcoma, although it remains a rare malignancy, with an incidence of 0.8–5 cases per million population per year. Early‐stage DFSP typically presents as non‐protuberant lesions, which gradually evolve into indurated, violaceous nodules. If left untreated, tumors can infiltrate deeper structures, including the fascia, muscles, periosteum, and bone. Distant metastatic lesions may develop over months to decades [9]. Clinically, DFSP is frequently misdiagnosed due to its resemblance to more common dermal conditions, with differential diagnoses including dermatofibroma, keloid, and morpheaform basal cell carcinoma [10]. A similar diagnostic challenge was observed in a rare case of DFSP affecting the middle finger of an infant, initially noticed as a lump at 3 months of age [11] (Figure 1). The indolent and asymptomatic course led to delayed medical evaluation and postponed diagnosis.

Histologically, DFSP is characterized by the proliferation of spindle cells extending into the subcutaneous fat, creating a distinctive honeycomb pattern. Definitive diagnosis is enhanced by immunohistochemical staining, as DFSP cells typically express CD34 and apolipoprotein D [4]. Conversely, immunohistochemical markers such as factor XIIIa, stromelysin III, CD44, CD163, and D2–40 are negative in DFSP [12]. One limitation of our case was the absence of molecular studies to confirm the presence of the COL1A1‐PDGFB fusion (Figure 1).

Histologically, DFSP exhibits multiple variants, including myxoid, pigmented, giant cell fibroblastoma, giant cell, sclerotic, granular cell, and fibrosarcomatous (FS) types. During tumor progression, spindle cells undergo differentiation, resulting in morphological heterogeneity among these variants. Although most variants share a similar clinical presentation, FS‐DFSP is distinct due to its association with increased local recurrence and metastatic potential. Eighty to ninety percent of DFSP variants that are not fibrosarcomatous are classified as conventional DFSP, whereas 10%–20% are FS‐DFSP, which carry a 5%–15% risk of metastasis [13] (Figure 1). In 2023, our patient developed the tumor for the fourth time, which was histologically confirmed as grade 2 fibrosarcomatous DFSP. Following surgical excision, no metastasis was detected.

When metastasis occurs, the lungs are the most common site, whereas nodal involvement is rare [14]. In 2024, a CT scan of the patient's abdomen revealed nodal metastasis, with enlarged lymph nodes along the right distal external iliac artery, as well as a few enhancing nodes in the inguinal and axillary regions.

The prognosis of DFSP is generally favorable, even in cases of distant metastasis. A recent population‐based retrospective cohort analysis by Chen, Shijing MD, examined 7567 patients with confirmed DFSP to evaluate survival outcomes and prognostic factors. The study reported that only 1.8% of patients died from DFSP, with mortality significantly higher in tumors of grade 3 and above. Patients with grade 3 tumors exhibited poor prognosis, while differences between grades 1 and 2 were negligible. Although DFSP demonstrates a high rate of local recurrence, distant metastases are uncommon, and overall patient survival is minimally affected [7]. Regional and distant recurrences remain rare [15].

Our case is particularly unusual, as the patient experienced recurrent DFSP at different locations without a history of local recurrence, which is typically more common. All lesions occurred on the trunk, the most frequent site for DFSP. This case is among the few reported instances demonstrating increased recurrence, with a notably short interval between episodes. The patient experienced five recurrences over 8 years, with initial lesions appearing in 2016, followed by recurrences in 2018, 2021, 2023, and 2024.

Surgical excision remains the primary treatment modality for DFSP. The two main procedures are wide local excision (WLE), the traditional method, and Mohs micrographic surgery (MMS), a more advanced technique. WLE involves three‐dimensional removal of the tumor‐bearing area, including the skin, subcutaneous tissue, and underlying fascia, with a 2–3 cm margin of normal tissue surrounding the tumor [16]. MMS, in contrast, utilizes horizontal slicing during resection, followed by frozen section analysis and immunohistochemical evaluation for CD34 to ensure clear margins [17]. In a recent comparative study by Sanabria et al., the mean recurrence rate for patients treated with WLE was 19%, whereas those treated with MMS experienced a recurrence rate of only 2%, demonstrating the superior efficacy of MMS in reducing tumor recurrence [18].

Similar to gastrointestinal stromal tumors (GISTs), a deep‐seated soft tissue neoplasm in which low‐risk cases are managed with complete resection [19], the management of DFSP primarily focuses on achieving clear surgical margins to minimize the risk of recurrence. Inadequate margins significantly increase the likelihood of local recurrence, making aggressive surgical resection a critical component of management. This principle has been highlighted in rare presentations, such as DFSP of the middle finger in an infant, where radical amputation was ultimately required to achieve clear margins and prevent recurrence [11].

DFSP is considered a radiosensitive tumor, and radiotherapy should be considered as an adjuvant treatment when positive margins persist following resection [16]. In our case, the patient underwent WLE for all five procedures. Despite the generally high local recurrence rate associated with WLE, the patient did not experience local recurrence. Our patient could not be offered Mohs Micrographic Surgery even after multiple failed WLE and FS transformation, primarily due to the constraints of a resource‐limited setting. Furthermore, adjuvant radiotherapy was not administered, as the patient was lost to follow‐up following surgical excision.

## Author Contributions

**Hamna Tariq:** conceptualization, data curation, investigation, project administration, supervision, validation, writing – original draft, writing – review and editing. **Hadiya Javed:** data curation, investigation, methodology, validation, writing – original draft, writing – review and editing. **Mohammed Hammad Jaber Amin:** project administration, resources.

## Funding

The authors have nothing to report.

## Consent

Written informed consent was obtained from the patient to publish this report in accordance with the journal's patient consent policy.

## Conflicts of Interest

The authors declare no conflicts of interest.

## Data Availability

The authors have nothing to report.

## References

[1] B. Mujtaba , F. Wang , A. Taher , et al., “Dermatofibrosarcoma Protuberans: Pathological and Imaging Review,” Current Problems in Diagnostic Radiology 50, no. 2 (2020): 236–240. 32620358 10.1067/j.cpradiol.2020.05.011

[2] A. Allen , C. Ahn , and O. P. Sangüeza , “Dermatofibrosarcoma Protuberans,” Dermatologic Clinics 37, no. 4 (2019): 483–488. 31466588 10.1016/j.det.2019.05.006

[3] A. E. Acosta and C. S. Vélez , “Dermatofibrosarcoma Protuberans,” Current Treatment Options in Oncology 18, no. 9 (2017): 56. 28795284 10.1007/s11864-017-0498-5

[4] C. Luu , J. L. Messina , A. S. Brohl , and V. K. Sondak , “Dermatofibrosarcoma Protuberans,” in Textbook of Uncommon Cancer, 5th ed. (Wiley‐Blackwell, 2017), 994–1001.

[5] S. X. Lim , A. Ramaiya , N. J. Levell , and Z. C. Venables , “Review of Dermatofibrosarcoma Protuberans,” Clinical and Experimental Dermatology 48, no. 4 (2022): 297–302, 10.1093/ced/llac111. 36630365

[6] J. Marcoval , C. Moreno‐Vílchez , C. Torrecilla‐Vall‐Llosera , et al., “Dermatofibrosarcoma Protuberans: A Study of 148 Patients,” Dermatology 240, no. 3 (2024): 487–493. 38228098 10.1159/000536172 PMC11168446

[7] S. Chen , L. Xiong , L. Zhao , Y. Li , and L. Li , “Survival Outcomes and Prognostic Factors of Dermatofibrosarcoma Protuberans: A Population‐Based Retrospective Cohort Analysis,” Dermatologic Surgery 49, no. 9 (2023): 825–831. 37384896 10.1097/DSS.0000000000003853 PMC10461715

[8] C. Sohrabi , G. Mathew , N. Maria , A. Kerwan , T. Franchi , and R. A. Agha , “The Scare 2023 Guideline: Updating Consensus Surgical Case Report (SCARE) Guidelines,” International Journal of Surgery 109, no. 5 (2023): 1136–1140. 37013953 10.1097/JS9.0000000000000373 PMC10389401

[9] S. Sheidaei , M. Salehi , F. Abedian Kenari , and H. R. Jafari , “Dermatofibrosarcoma Protuberans Challenges: A Case Series and Review of the Literature,” Journal of Medical Case Reports 17, no. 1 (2023): 18. 36653860 10.1186/s13256-022-03728-6 PMC9850584

[10] A. Oliveira , E. Arzberger , I. Zalaudek , and R. Hofmann‐Wellenhof , “Diagnosis of Dermatofibrosarcoma Protuberans and Assessment of Pre‐Surgical Margins Using High‐Definition Optical Coherence Tomography Imaging,” Journal of the European Academy of Dermatology and Venereology 30, no. 4 (2016): 710–711. 25683002 10.1111/jdv.13007

[11] M. Hanna , A. S. A. Alkhatib , R. Alassri , R. Awada , D. Daboura , and N. Martini , “A Challenging Diagnosis of Dermatofibrosarcoma Protuberans of the Middle Finger in an Infant: A Case Report,” International Journal of Surgery Case Reports 120 (2024): 109890. 38865945 10.1016/j.ijscr.2024.109890 PMC11258620

[12] N. Larbcharoensub , J. Kayankarnnavee , S. Sanpaphant , K. Kiranantawat , C. Wirojtananugoon , and V. Sirikulchayanonta , “Clinicopathological Features of Dermatofibrosarcoma Protuberans,” Oncology Letters 11, no. 1 (2015): 661–667. 26870263 10.3892/ol.2015.3966 PMC4726970

[13] X. Hao , S. D. Billings , F. Wu , et al., “Dermatofibrosarcoma Protuberans: Update on the Diagnosis and Treatment,” Journal of Clinical Medicine 9, no. 6 (2020): 1752. 32516921 10.3390/jcm9061752 PMC7355835

[14] K. Thway , J. Noujaim , R. L. Jones , and C. Fisher , “Dermatofibrosarcoma Protuberans: Pathology, Genetics, and Potential Therapeutic Strategies,” Annals of Diagnostic Pathology 25 (2016): 64–71. 27806849 10.1016/j.anndiagpath.2016.09.013

[15] E. J. Rutgers , B. B. Kroon , C. E. Albus‐Lutter , and E. Gortzak , “Dermatofibrosarcoma Protuberans: Treatment and Prognosis,” European Journal of Surgical Oncology 18, no. 3 (1992): 241–248. 1607035

[16] R. Hamid , A. Hafeez , A. M. Darzi , I. Zaroo , H. Owais , and A. Akhter , “Dermatofibrosarcoma Protuberans: Role of Wide Local Excision,” South Asian Journal of Cancer 2, no. 4 (2013): 232–238. 24455646 10.4103/2278-330X.119926 PMC3889049

[17] J. Brooks and M. L. Ramsey , Cancer, Dermatofibrosarcoma Protuberans (StatPearls Publishing, 2019), https://www.ncbi.nlm.nih.gov/books/NBK513305/. 30020677

[18] A. Sanabria , P. Pinillos , C. Chiesa‐Estomba , et al., “Comparing Mohs Micrographic Surgery and Wide Local Excision in the Management of Head and Neck Dermatofibrosarcoma Protuberans: A Scoping Review,” Journal of Dermatological Treatment 35, no. 1 (2023): 2295816. 38146660 10.1080/09546634.2023.2295816

[19] E. Alabdallah , M. H. D. M. Al Mouallem , B. Al‐Ghotani , N. Martini , and S. Al‐Mahasna , “Retroperitoneal Extra Gastrointestinal Stromal Tumor: A Case Report,” International Journal of Surgery Case Reports 108 (2023): 108442. 37392585 10.1016/j.ijscr.2023.108442 PMC10382849

